# Crystal structure of *N*-(1-allyl-3-chloro-4-eth­oxy-1*H*-indazol-5-yl)-4-meth­oxybenzene­sulfonamide

**DOI:** 10.1107/S1600536814018492

**Published:** 2014-08-20

**Authors:** Hakima Chicha, El Mostapha Rakib, Latifa Bouissane, Mohamed Saadi, Lahcen El Ammari

**Affiliations:** a Laboratoire de Chimie Organique et Analytique, Université Sultan Moulay Slimane, Faculté des Sciences et Techniques, Béni-Mellal, BP 523, Morocco; b Laboratoire de Chimie du Solide Appliquée, Faculté des Sciences, Université Mohammed V-Agdal, Avenue Ibn Battouta, BP 1014, Rabat, Morocco

**Keywords:** crystal structure, indazole, benzene­sulfonamide, hydrogen bonds

## Abstract

In the title compound, C_19_H_20_ClN_3_O_4_S, the benzene ring is inclined to the indazole ring system (r.m.s. deviation = 0.014 Å) by 65.07 (8)°. The allyl and eth­oxy groups are almost normal to the indazole ring, as indicated by the respective torsion angles [N—N—C—C = 111.6 (2) and C—C—O—C = −88.1 (2)°]. In the crystal, mol­ecules are connected by N—H⋯N hydrogen bonds, forming helical chains propagating along [010]. The chains are linked by C—H⋯O hydrogen bonds, forming a three-dimensional network.

## Related literature   

For the biological activity of sulfonamides, see: El-Sayed *et al.* (2011 ▶); Mustafa *et al.* (2012 ▶); Bouissane *et al.* (2006 ▶); Ghorab *et al.* (2009 ▶). For similar compounds, see: Abbassi *et al.* (2012 ▶, 2013 ▶); Chicha *et al.* (2014 ▶).
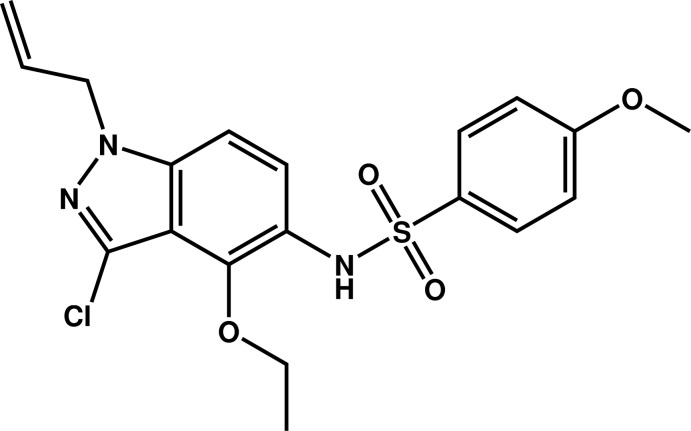


## Experimental   

### Crystal data  

C_19_H_20_ClN_3_O_4_S
*M* *_r_* = 421.89
Monoclinic, 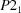

*a* = 8.2699 (7) Å
*b* = 13.1235 (12) Å
*c* = 10.0026 (9) Å
β = 110.379 (5)°
*V* = 1017.64 (16) Å^3^
*Z* = 2
Mo *K*α radiation
μ = 0.32 mm^−1^
*T* = 296 K
0.42 × 0.32 × 0.28 mm

### Data collection  

Bruker X8 APEX Diffractometer
Absorption correction: multi-scan (*SADABS*; Sheldrick, 2008 ▶) *T*_min_ = 0.670, *T*_max_ = 0.746
12792 measured reflections
5605 independent reflections
4754 reflections with *I* > 2σ(*I*)
*R*_int_ = 0.028

### Refinement  

*R*[*F* ^2^ > 2σ(*F* ^2^)] = 0.035
*wR*(*F* ^2^) = 0.088
*S* = 1.03
5605 reflections
253 parameters
1 restraint
H-atom parameters constrained
Δρ_max_ = 0.18 e Å^−3^
Δρ_min_ = −0.20 e Å^−3^
Absolute structure: Flack & Bernardinelli (2000 ▶)
Absolute structure parameter: −0.04 (4)

### 

Data collection: *APEX2* (Bruker, 2009 ▶); cell refinement: *SAINT* (Bruker, 2009 ▶); data reduction: *SAINT*; program(s) used to solve structure: *SHELXS97* (Sheldrick, 2008 ▶); program(s) used to refine structure: *SHELXL97* (Sheldrick, 2008 ▶); molecular graphics: *ORTEP-3 for Windows* (Farrugia, 2012 ▶); software used to prepare material for publication: *PLATON* (Spek, 2009 ▶) and *publCIF* (Westrip, 2010 ▶).

## Supplementary Material

Crystal structure: contains datablock(s) I. DOI: 10.1107/S1600536814018492/su2771sup1.cif

Structure factors: contains datablock(s) I. DOI: 10.1107/S1600536814018492/su2771Isup2.hkl

Supporting information file. DOI: 10.1107/S1600536814018492/su2771Isup3.cml

**Fig. 1.**
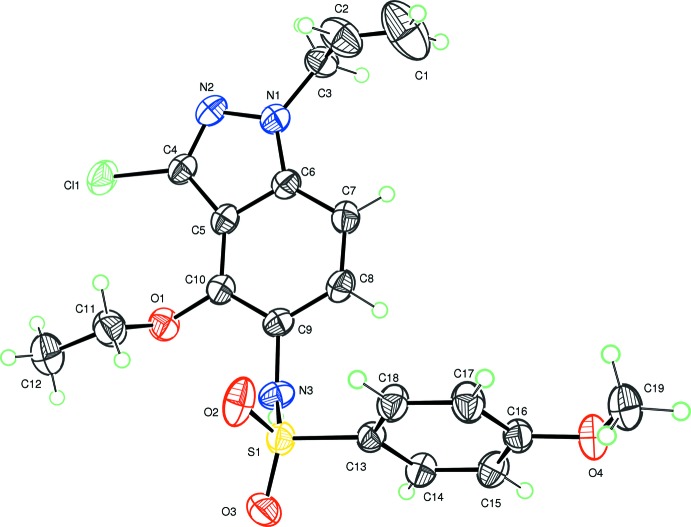
A view of the molecular structure of the title molecule, with atom labelling. Displacement ellipsoids are drawn at the 50% probability level.

. DOI: 10.1107/S1600536814018492/su2771fig1.tif
A view of the mol­ecular structure of the title mol­ecule, with atom labelling. Displacement ellipsoids are drawn at the 50% probability level.

**Fig. 2.**
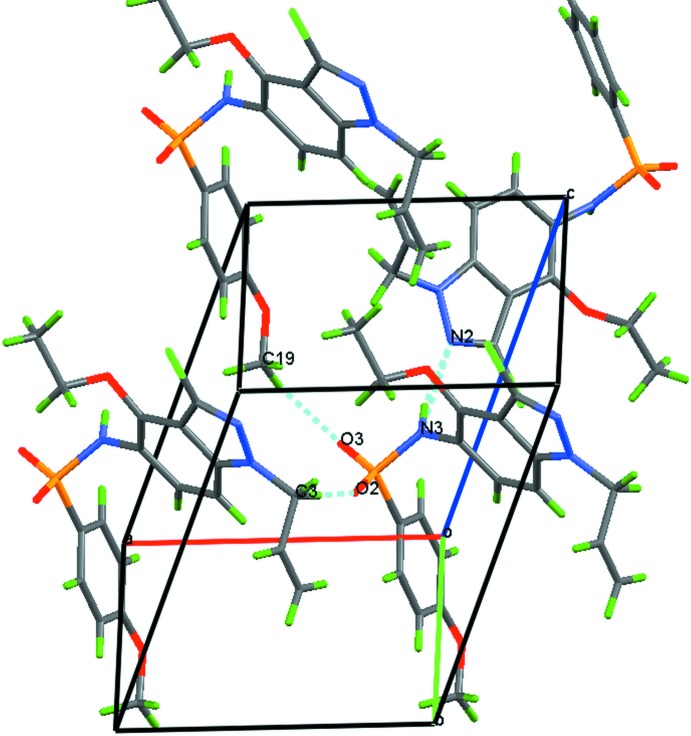
A partial view of the crystal packing of the title compound. The hydrogen bonds are shown as dashed lines (see Table 1 for details).

. DOI: 10.1107/S1600536814018492/su2771fig2.tif
A partial view of the crystal packing of the title compound. The hydrogen bonds are shown as dashed lines (see Table 1 for details).

CCDC reference: 1019238

Additional supporting information:  crystallographic information; 3D view; checkCIF report

## Figures and Tables

**Table 1:** Hydrogen-bond geometry (Å, °)

*D*—H⋯*A*	*D*—H	H⋯*A*	*D*⋯*A*	*D*—H⋯*A*
N3—H1*N*⋯N2^i^	0.84	2.11	2.931 (2)	166
C19—H19*A*⋯O3^ii^	0.96	2.37	3.285 (2)	159
C3—H3*B*⋯O2^iii^	0.97	2.47	3.418 (3)	165

Symmetry codes: (i) 
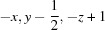
; (ii) 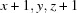; (iii) 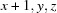.

## supplementary crystallographic information

### S1. Experimental

A mixture of 1-allyl-3-chloro-5-nitroindazole (1.22 mmol) and anhydrous SnCl_2_ (1.1 g, 6.1 mmol) in 25 ml of absolute ethanol was heated at 333 K for 6 h. After reduction, the starting material disappeared, and the solution was allowed to cool. The pH was made slightly basic (pH 7–8) by addition of 5% aqueous potassium bicarbonate before extraction with ethyl acetate. The organic phase was washed with brine and dried over magnesium sulfate. The solvent was removed to afford the amine, which was immediately dissolved in pyridine (5 ml) and then reacted with 4-methoxybenzenesulfonyl chloride (1.25 mmol) at room temperature for 24 h. After the reaction mixture was concentrated *in vacuo*, the resulting residue was purified by flash chromatography (eluted with ethyl acetate:hexane 2:8). Crystals of the title compound were obtained by recrystallization from ethanol (yield = 43%; m.p. = 397 K).

### S2. Refinement

Reflections (001) and (100), affected by the beam stop, were removed from the refinement. The H atoms were located in a difference map and treated as riding atoms: N–H = 0.84 Å, C–H = 0.93 - 0.97 Å, with *U*_iso_(H) = 1.5*U*_eq_(C-methyl) and = 1.2*U*_eq_(N,C) for other H atoms.

### Crystal data

**Table:**

C_19_H_20_ClN_3_O_4_S	*F*(000) = 440
*M**_r_* = 421.89	*D*_x_ = 1.377 Mg m^−^^3^
Monoclinic, *P*2_1_	Melting point: 397 K
Hall symbol: P 2yb	Mo *K*α radiation, λ = 0.71073 Å
*a* = 8.2699 (7) Å	Cell parameters from 5605 reflections
*b* = 13.1235 (12) Å	θ = 2.6–29.6°
*c* = 10.0026 (9) Å	µ = 0.32 mm^−^^1^
β = 110.379 (5)°	*T* = 296 K
*V* = 1017.64 (16) Å^3^	Prism, colourless
*Z* = 2	0.42 × 0.32 × 0.28 mm

### Data collection

**Table:**

Bruker X8 APEX Diffractometer	5605 independent reflections
Radiation source: fine-focus sealed tube	4754 reflections with *I* > 2σ(*I*)
Graphite monochromator	*R*_int_ = 0.028
φ and ω scans	θ_max_ = 29.6°, θ_min_ = 2.6°
Absorption correction: multi-scan (*SADABS*; Sheldrick, 2008)	*h* = −11→11
*T*_min_ = 0.670, *T*_max_ = 0.746	*k* = −18→18
12792 measured reflections	*l* = −12→13

### Refinement

**Table:**

Refinement on *F*^2^	Secondary atom site location: difference Fourier map
Least-squares matrix: full	Hydrogen site location: inferred from neighbouring sites
*R*[*F*^2^ > 2σ(*F*^2^)] = 0.035	H-atom parameters constrained
*wR*(*F*^2^) = 0.088	*w* = 1/[σ^2^(*F*_o_^2^) + (0.0435*P*)^2^ + 0.0481*P*] where *P* = (*F*_o_^2^ + 2*F*_c_^2^)/3
*S* = 1.03	(Δ/σ)_max_ = 0.001
5605 reflections	Δρ_max_ = 0.18 e Å^−^^3^
253 parameters	Δρ_min_ = −0.20 e Å^−^^3^
1 restraint	Absolute structure: Flack & Bernardinelli (2000)
Primary atom site location: structure-invariant direct methods	Absolute structure parameter: −0.04 (4)

### Special details

**Table:**

Geometry. All e.s.d.'s (except the e.s.d. in the dihedral angle between two l.s. planes) are estimated using the full covariance matrix. The cell e.s.d.'s are taken into account individually in the estimation of e.s.d.'s in distances, angles and torsion angles; correlations between e.s.d.'s in cell parameters are only used when they are defined by crystal symmetry. An approximate (isotropic) treatment of cell e.s.d.'s is used for estimating e.s.d.'s involving l.s. planes.
Refinement. Refinement of *F*^2^ against all reflections. The weighted *R*-factor *wR* and goodness of fit *S* are based on *F*^2^, conventional *R*-factors *R* are based on *F*, with *F* set to zero for negative *F*^2^. The threshold expression of *F*^2^ > σ(*F*^2^) is used only for calculating *R*-factors(gt) *etc*. and is not relevant to the choice of reflections for refinement. *R*-factors based on *F*^2^ are statistically about twice as large as those based on *F*, and *R*- factors based on all data will be even larger.

### Fractional atomic coordinates and isotropic or equivalent isotropic displacement parameters (Å^2^)

**Table:**

	*x*	*y*	*z*	*U*_iso_*/*U*_eq_	
C1	0.5375 (4)	0.8680 (4)	0.9417 (3)	0.1124 (13)	
H1A	0.6182	0.8159	0.9577	0.135*	
H1B	0.5344	0.9084	1.0173	0.135*	
C2	0.4301 (3)	0.8844 (2)	0.8151 (3)	0.0706 (7)	
H2	0.3506	0.9370	0.8018	0.085*	
C3	0.4268 (2)	0.82350 (16)	0.6893 (2)	0.0519 (4)	
H3A	0.4629	0.8663	0.6257	0.062*	
H3B	0.5089	0.7680	0.7206	0.062*	
C4	0.0173 (2)	0.76621 (13)	0.43723 (18)	0.0438 (4)	
C5	0.0066 (2)	0.69652 (13)	0.54239 (16)	0.0396 (3)	
C6	0.1670 (2)	0.70913 (13)	0.65413 (18)	0.0411 (4)	
C7	0.2124 (2)	0.65003 (15)	0.77993 (19)	0.0472 (4)	
H7	0.3187	0.6575	0.8526	0.057*	
C8	0.0936 (2)	0.58180 (14)	0.78968 (19)	0.0455 (4)	
H8	0.1200	0.5420	0.8714	0.055*	
C9	−0.0697 (2)	0.56870 (13)	0.68005 (17)	0.0387 (3)	
C10	−0.1141 (2)	0.62525 (13)	0.55548 (17)	0.0389 (3)	
C11	−0.4058 (3)	0.67175 (17)	0.4348 (2)	0.0595 (5)	
H11A	−0.3680	0.7422	0.4467	0.071*	
H11B	−0.4498	0.6548	0.5102	0.071*	
C12	−0.5450 (3)	0.6575 (2)	0.2914 (3)	0.0826 (8)	
H12A	−0.6410	0.7009	0.2846	0.124*	
H12B	−0.5821	0.5877	0.2807	0.124*	
H12C	−0.5006	0.6748	0.2176	0.124*	
C13	−0.1432 (2)	0.47263 (13)	0.97527 (17)	0.0372 (3)	
C14	−0.0709 (2)	0.37584 (14)	1.00480 (19)	0.0445 (4)	
H14	−0.0998	0.3261	0.9342	0.053*	
C15	0.0440 (2)	0.35386 (14)	1.1395 (2)	0.0472 (4)	
H15	0.0936	0.2895	1.1594	0.057*	
C16	0.0854 (2)	0.42823 (14)	1.24556 (18)	0.0412 (4)	
C17	0.0108 (3)	0.52403 (15)	1.21696 (19)	0.0509 (5)	
H17	0.0365	0.5732	1.2882	0.061*	
C18	−0.1031 (2)	0.54584 (15)	1.08055 (19)	0.0472 (4)	
H18	−0.1525	0.6103	1.0603	0.057*	
C19	0.2358 (3)	0.4703 (2)	1.4897 (2)	0.0654 (6)	
H19A	0.3188	0.4414	1.5739	0.098*	
H19B	0.1310	0.4845	1.5071	0.098*	
H19C	0.2808	0.5323	1.4656	0.098*	
N1	0.25612 (19)	0.78146 (13)	0.61078 (16)	0.0475 (3)	
H1N	−0.1966	0.4399	0.6454	0.057*	
N2	0.1641 (2)	0.81619 (12)	0.47747 (17)	0.0487 (4)	
N3	−0.18652 (19)	0.49314 (12)	0.69400 (14)	0.0450 (3)	
O1	−0.26451 (16)	0.60654 (10)	0.44257 (13)	0.0492 (3)	
O2	−0.3468 (2)	0.60356 (12)	0.80661 (15)	0.0631 (4)	
O3	−0.41372 (18)	0.41918 (13)	0.76263 (15)	0.0663 (4)	
O4	0.20127 (17)	0.39973 (12)	1.37445 (14)	0.0575 (4)	
S1	−0.29029 (5)	0.50045 (3)	0.80328 (4)	0.04255 (11)	
Cl1	−0.13391 (7)	0.78969 (4)	0.27168 (5)	0.06011 (14)	

### Atomic displacement parameters (Å^2^)

**Table:**

	*U*^11^	*U*^22^	*U*^33^	*U*^12^	*U*^13^	*U*^23^
C1	0.0692 (16)	0.197 (4)	0.0710 (18)	−0.008 (2)	0.0241 (14)	−0.035 (2)
C2	0.0470 (11)	0.0776 (16)	0.0869 (16)	−0.0028 (11)	0.0230 (11)	−0.0236 (13)
C3	0.0415 (9)	0.0562 (11)	0.0615 (11)	−0.0034 (8)	0.0223 (8)	0.0045 (9)
C4	0.0545 (10)	0.0401 (10)	0.0375 (8)	0.0026 (8)	0.0168 (7)	0.0043 (7)
C5	0.0474 (9)	0.0380 (8)	0.0345 (7)	0.0047 (7)	0.0155 (6)	0.0034 (6)
C6	0.0436 (8)	0.0399 (9)	0.0415 (8)	0.0019 (7)	0.0169 (7)	0.0041 (7)
C7	0.0441 (9)	0.0529 (11)	0.0404 (8)	0.0016 (8)	0.0096 (7)	0.0075 (7)
C8	0.0517 (10)	0.0480 (10)	0.0367 (8)	0.0037 (8)	0.0154 (7)	0.0096 (7)
C9	0.0471 (9)	0.0340 (8)	0.0377 (8)	−0.0011 (7)	0.0184 (7)	−0.0012 (6)
C10	0.0460 (8)	0.0355 (8)	0.0343 (7)	0.0020 (7)	0.0129 (6)	−0.0020 (6)
C11	0.0527 (10)	0.0579 (12)	0.0599 (11)	0.0003 (10)	0.0097 (9)	−0.0046 (10)
C12	0.0651 (14)	0.0873 (19)	0.0745 (15)	−0.0002 (13)	−0.0023 (12)	0.0097 (14)
C13	0.0374 (7)	0.0407 (9)	0.0341 (7)	0.0006 (6)	0.0129 (6)	0.0056 (6)
C14	0.0526 (9)	0.0398 (9)	0.0398 (8)	0.0039 (8)	0.0145 (7)	−0.0014 (7)
C15	0.0530 (10)	0.0396 (9)	0.0487 (9)	0.0108 (8)	0.0171 (8)	0.0061 (7)
C16	0.0353 (7)	0.0475 (10)	0.0390 (8)	0.0030 (7)	0.0108 (6)	0.0053 (7)
C17	0.0548 (10)	0.0466 (11)	0.0424 (9)	0.0043 (8)	0.0057 (7)	−0.0067 (7)
C18	0.0532 (10)	0.0382 (9)	0.0447 (9)	0.0072 (7)	0.0100 (8)	0.0024 (7)
C19	0.0630 (12)	0.0784 (16)	0.0416 (10)	−0.0014 (11)	0.0013 (8)	0.0019 (10)
N1	0.0483 (7)	0.0480 (8)	0.0464 (8)	−0.0015 (7)	0.0169 (6)	0.0078 (7)
N2	0.0601 (9)	0.0433 (8)	0.0465 (8)	0.0024 (7)	0.0234 (7)	0.0091 (6)
N3	0.0608 (8)	0.0372 (7)	0.0419 (7)	−0.0087 (7)	0.0241 (6)	−0.0053 (6)
O1	0.0551 (7)	0.0475 (7)	0.0390 (6)	−0.0016 (6)	0.0089 (5)	−0.0066 (5)
O2	0.0731 (9)	0.0687 (10)	0.0497 (8)	0.0340 (8)	0.0242 (7)	0.0146 (7)
O3	0.0477 (7)	0.0917 (12)	0.0499 (8)	−0.0213 (8)	0.0049 (6)	0.0132 (8)
O4	0.0520 (7)	0.0689 (9)	0.0419 (6)	0.0142 (7)	0.0041 (5)	0.0047 (6)
S1	0.03950 (19)	0.0505 (2)	0.03584 (18)	0.00353 (19)	0.01090 (14)	0.00789 (17)
Cl1	0.0752 (3)	0.0580 (3)	0.0390 (2)	−0.0010 (2)	0.0097 (2)	0.0121 (2)

### Geometric parameters (Å, º)

**Table:**

C1—C2	1.287 (4)	C11—H11B	0.9700
C1—H1A	0.9300	C12—H12A	0.9600
C1—H1B	0.9300	C12—H12B	0.9600
C2—C3	1.483 (3)	C12—H12C	0.9600
C2—H2	0.9300	C13—C18	1.378 (3)
C3—N1	1.463 (2)	C13—C14	1.391 (2)
C3—H3A	0.9700	C13—S1	1.7650 (16)
C3—H3B	0.9700	C14—C15	1.382 (2)
C4—N2	1.313 (2)	C14—H14	0.9300
C4—C5	1.420 (2)	C15—C16	1.393 (3)
C4—Cl1	1.7201 (17)	C15—H15	0.9300
C5—C10	1.407 (2)	C16—O4	1.3636 (19)
C5—C6	1.416 (2)	C16—C17	1.386 (3)
C6—N1	1.362 (2)	C17—C18	1.391 (2)
C6—C7	1.413 (2)	C17—H17	0.9300
C7—C8	1.358 (3)	C18—H18	0.9300
C7—H7	0.9300	C19—O4	1.428 (3)
C8—C9	1.423 (2)	C19—H19A	0.9600
C8—H8	0.9300	C19—H19B	0.9600
C9—C10	1.385 (2)	C19—H19C	0.9600
C9—N3	1.425 (2)	N1—N2	1.363 (2)
C10—O1	1.3801 (19)	N3—S1	1.6106 (14)
C11—O1	1.428 (2)	N3—H1N	0.8390
C11—C12	1.506 (3)	O2—S1	1.4358 (15)
C11—H11A	0.9700	O3—S1	1.4338 (15)
			
C2—C1—H1A	120.0	C11—C12—H12C	109.5
C2—C1—H1B	120.0	H12A—C12—H12C	109.5
H1A—C1—H1B	120.0	H12B—C12—H12C	109.5
C1—C2—C3	123.2 (3)	C18—C13—C14	120.21 (15)
C1—C2—H2	118.4	C18—C13—S1	120.10 (13)
C3—C2—H2	118.4	C14—C13—S1	119.68 (13)
N1—C3—C2	112.82 (16)	C15—C14—C13	119.75 (16)
N1—C3—H3A	109.0	C15—C14—H14	120.1
C2—C3—H3A	109.0	C13—C14—H14	120.1
N1—C3—H3B	109.0	C14—C15—C16	119.95 (16)
C2—C3—H3B	109.0	C14—C15—H15	120.0
H3A—C3—H3B	107.8	C16—C15—H15	120.0
N2—C4—C5	112.59 (15)	O4—C16—C17	124.04 (16)
N2—C4—Cl1	119.34 (13)	O4—C16—C15	115.67 (16)
C5—C4—Cl1	128.07 (14)	C17—C16—C15	120.29 (15)
C10—C5—C6	120.30 (15)	C16—C17—C18	119.33 (17)
C10—C5—C4	136.61 (15)	C16—C17—H17	120.3
C6—C5—C4	103.08 (15)	C18—C17—H17	120.3
N1—C6—C7	131.36 (16)	C13—C18—C17	120.43 (17)
N1—C6—C5	107.00 (15)	C13—C18—H18	119.8
C7—C6—C5	121.61 (16)	C17—C18—H18	119.8
C8—C7—C6	116.83 (15)	O4—C19—H19A	109.5
C8—C7—H7	121.6	O4—C19—H19B	109.5
C6—C7—H7	121.6	H19A—C19—H19B	109.5
C7—C8—C9	122.74 (16)	O4—C19—H19C	109.5
C7—C8—H8	118.6	H19A—C19—H19C	109.5
C9—C8—H8	118.6	H19B—C19—H19C	109.5
C10—C9—C8	120.76 (16)	C6—N1—N2	111.39 (14)
C10—C9—N3	119.00 (15)	C6—N1—C3	128.29 (16)
C8—C9—N3	120.16 (14)	N2—N1—C3	120.30 (16)
O1—C10—C9	121.44 (16)	C4—N2—N1	105.93 (14)
O1—C10—C5	120.56 (15)	C9—N3—S1	124.38 (12)
C9—C10—C5	117.75 (15)	C9—N3—H1N	117.2
O1—C11—C12	108.46 (19)	S1—N3—H1N	118.3
O1—C11—H11A	110.0	C10—O1—C11	115.14 (13)
C12—C11—H11A	110.0	C16—O4—C19	117.56 (16)
O1—C11—H11B	110.0	O3—S1—O2	120.14 (10)
C12—C11—H11B	110.0	O3—S1—N3	104.96 (9)
H11A—C11—H11B	108.4	O2—S1—N3	109.07 (8)
C11—C12—H12A	109.5	O3—S1—C13	107.67 (8)
C11—C12—H12B	109.5	O2—S1—C13	106.96 (8)
H12A—C12—H12B	109.5	N3—S1—C13	107.45 (8)

### Hydrogen-bond geometry (Å, º)

**Table:**

*D*—H···*A*	*D*—H	H···*A*	*D*···*A*	*D*—H···*A*
N3—H1*N*···N2^i^	0.84	2.11	2.931 (2)	166
C19—H19*A*···O3^ii^	0.96	2.37	3.285 (2)	159
C3—H3*B*···O2^iii^	0.97	2.47	3.418 (3)	165

Symmetry codes: (i) −*x*, *y*−1/2, −*z*+1; (ii) *x*+1, *y*, *z*+1; (iii) *x*+1, *y*, *z*.
