# Supplementary figures and images for: Crystal structure of N-(1-allyl-3-chloro-4-eth­oxy-1H-indazol-5-yl)-4-meth­oxybenzene­sulfonamide
Source: Acta Crystallogr Sect E Struct Rep Online. 2014 Aug 20;70(Pt 9):o1029–30. doi: 10.1107/S1600536814018492 (PMC4186176; doi:10.1107/S1600536814018492)

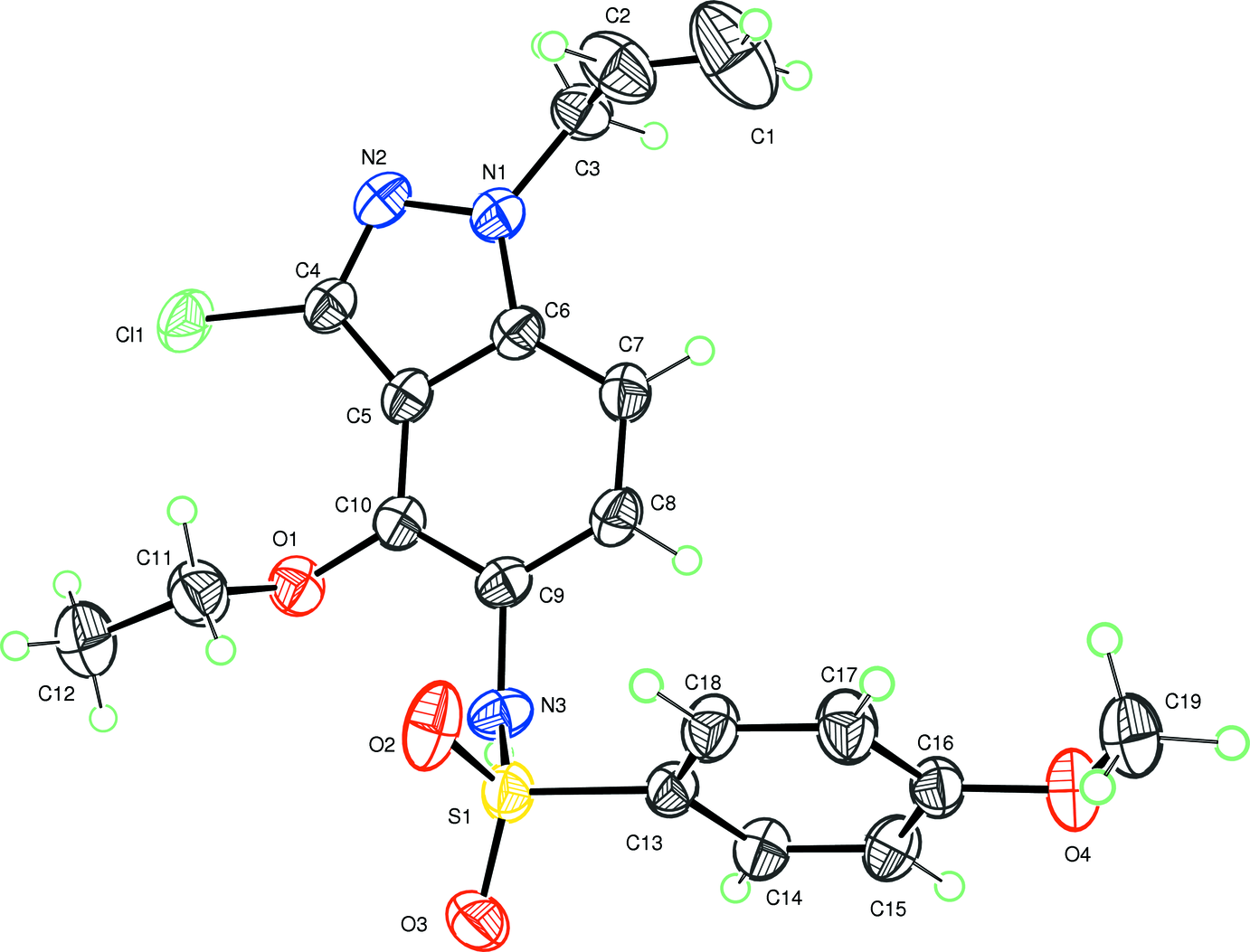

Supplement: Supplementary file 4 [file e-70-o1029-fig1.tif]

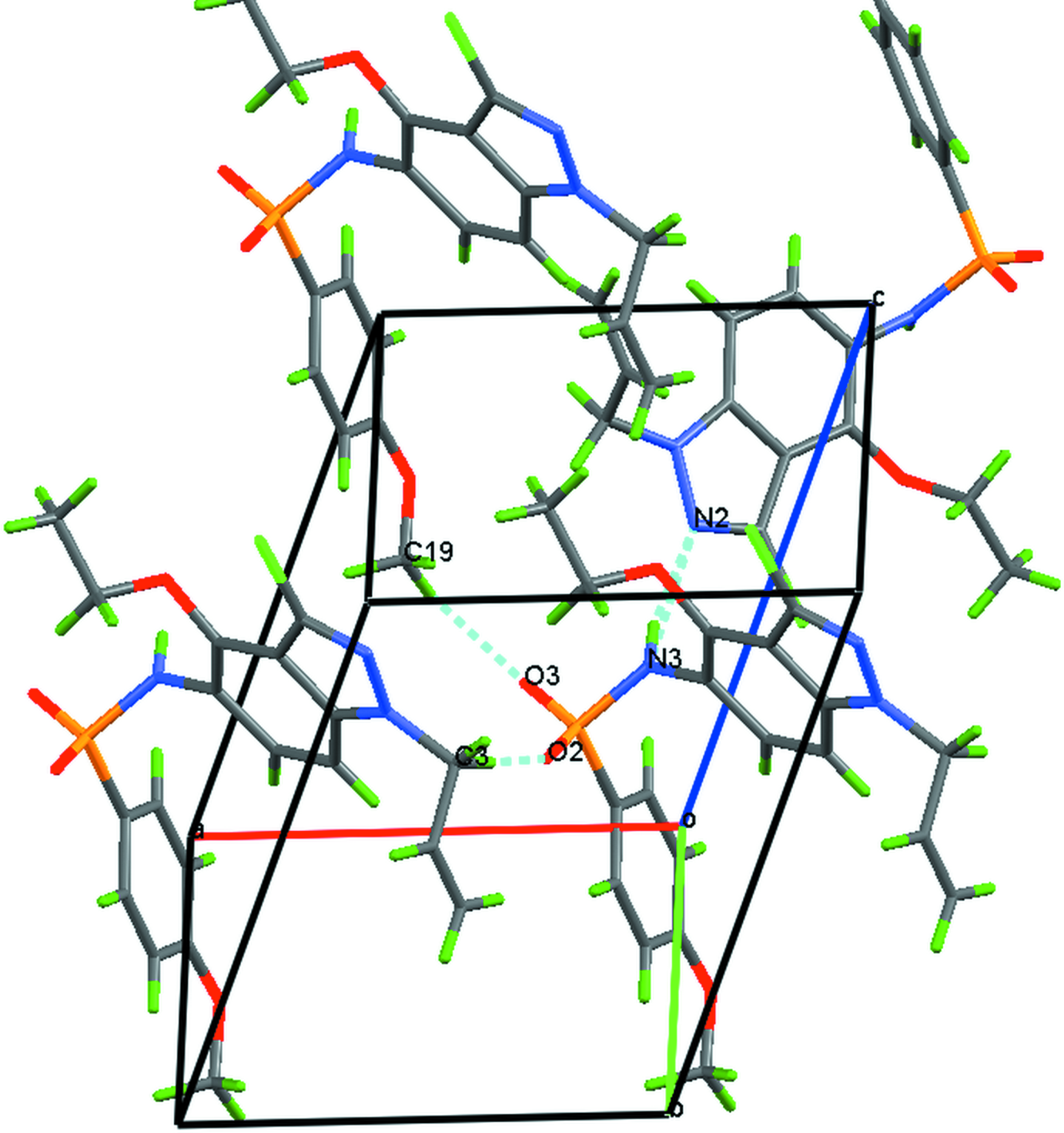

Supplement: Supplementary file 5 [file e-70-o1029-fig2.tif]
